# Supplementary figures and images for: Comprehensive analysis of immune-related genes reveals diagnostic biomarkers and molecular subtypes in diabetic retinopathy
Source: PLoS One. 2026 Apr 22;21(4):e0346725. doi: 10.1371/journal.pone.0346725 (PMC13102194; doi:10.1371/journal.pone.0346725)

Control

DM

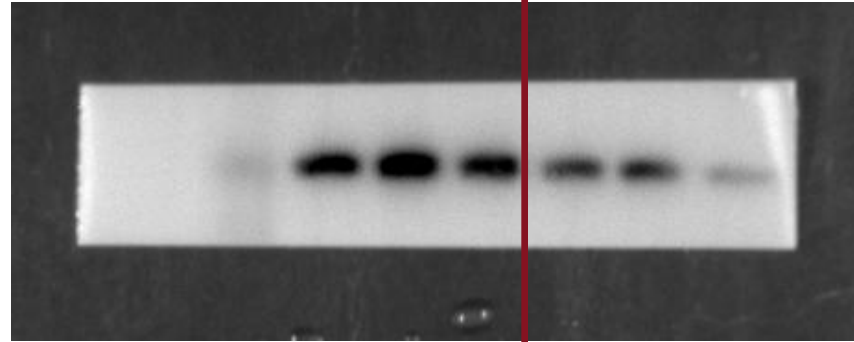

VGF

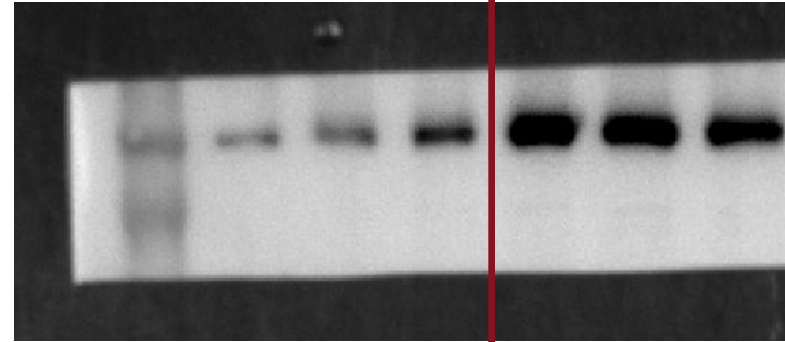

PLAU

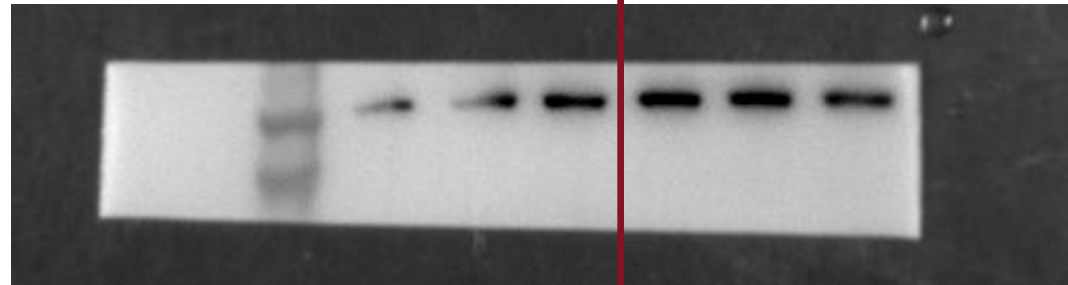

PLAUR

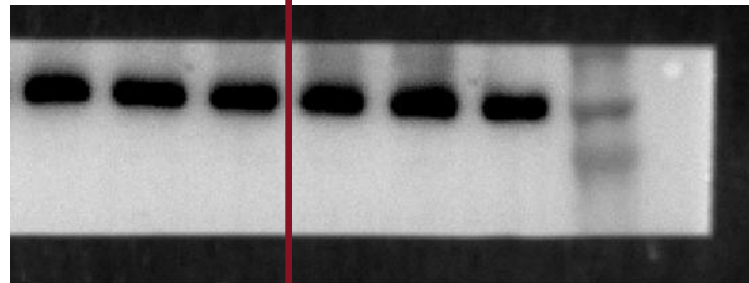

GAPDH

Supplement: S1 File — The file contains all original Western blot images underlying the results presented in Figure 9. Each image is labeled with sample loading order, molecular weight markers, and the corresponding figure panel. (PDF) [file pone.0346725.s001.pdf]
