# Supplementary material for: Comprehensive analysis of immune-related genes reveals diagnostic biomarkers and molecular subtypes in diabetic retinopathy
Source: PLoS One. 2026 Apr 22;21(4):e0346725. doi: 10.1371/journal.pone.0346725 (PMC13102194; doi:10.1371/journal.pone.0346725)
Supplement: Attachment — Submitted filename: Response to Reviewers.docx [file pone.0346725.s003.docx]

**Response to Reviewers**

Manuscript ID: PONE-D-25-66057

Title: Comprehensive Analysis of Immune-Related Genes Reveals Diagnostic Biomarkers and Molecular Subtypes in Diabetic Retinopathy

Journal: PLOS ONE

Dear Dr. Chaurasia and Reviewers,

Thank you very much for your thoughtful and constructive comments on our manuscript entitled "Comprehensive Analysis of Immune-Related Genes Reveals Diagnostic Biomarkers and Molecular Subtypes in Diabetic Retinopathy" (PONE-D-25-66057). We have carefully considered all the suggestions and have revised the manuscript accordingly. Below, we provide a point-by-point response to each comment. All changes in the manuscript have been marked using the "Track Changes" feature for your convenience.

We sincerely hope that the revised manuscript now meets the publication standards of PLOS ONE.

Sincerely,

Xuejun Wang, PhD

Xingtao Zhou, PhD

Corresponding Authors

**Response to Journal Requirements**

**Requirement 1: PLOS ONE Style Requirements**

**Comment:** Please ensure that your manuscript meets PLOS ONE's style requirements, including those for file naming.

**Response:** We have carefully reviewed the PLOS ONE formatting guidelines and made the following adjustments:

- All figures are now cited as "Fig 1", "Fig 2", etc. throughout the text, as required (previously "Figure 1").

- Multiple figures are cited as "Figs 1 and 2" or "Figs 1-3" where appropriate.

- The manuscript has been formatted with Times New Roman font, double-spacing, and proper paragraph indentation.

- Figure titles and legends have been revised to be more descriptive and follow the required format.

**Requirement 2: Animal Experiment Details**

**Comment:** Please provide additional information regarding the experiments involving animals, including methods of sacrifice, methods of anesthesia, and efforts to alleviate suffering.

**Response:** Thank you for this reminder. We have already included these details in the original submission. In Section 2.12, we explicitly state: "Mice were anesthetized with intraperitoneal sodium pentobarbital (50 mg/kg) prior to tissue collection. Euthanasia was performed by cervical dislocation under deep anesthesia. All efforts were made to minimize animal suffering." We have also added the ethical approval number (No. P 2022026) as required.

**Requirement 3: Funding Disclosure**

**Comment:** Please state what role the funders took in the study.

**Response:** We have prepared the following statement to be included in the cover letter:

> "The funders had no role in study design, data collection and analysis, decision to publish, or preparation of the manuscript."

We will ensure this is added to the submission system.

**Requirement 4: Funding Information in Manuscript**

**Comment:** Please remove any funding-related text from the manuscript.

**Response:** We have removed the "Funding" section from the manuscript as requested. The funding information will only appear in the online submission form.

**Requirement 5: Data Availability Statement**

**Comment:** The data contact point must not be an author and should be an institutional contact.

**Response:** Thank you for your guidance regarding our data availability statement. We have carefully reviewed the PLOS ONE data policy and would like to clarify the following:

Primary Data Source: The main data used in this study are publicly available from the Gene Expression Omnibus (GEO) database under accession numbers GSE60436 and GSE102485. Full citations for these datasets are provided in the References section ([13] and [14]).

---

**Response to Reviewer 1**

General Comment: The study provides a comprehensive analysis of immune-related genes in DR, identifying IL10RA, PLAUR, PLAU, VTN, and VGF as potential biomarkers with validation in an STZ-induced mouse model.

Response: Thank you for your positive assessment of our work. We appreciate your recognition of the comprehensive approach we have taken.

**Comment 1: Inclusion Criteria for GEO Datasets**

**Comment:** List out the inclusion criteria for selecting the two GEO datasets used in the study.

**Response:** Thank you for this suggestion. We have added the inclusion criteria in Section 2.1:

> "The inclusion criteria for dataset selection were as follows: (1) retinal tissue samples from Homo sapiens; (2) clearly defined DR and normal control groups; (3) availability of complete expression data; and (4) sample size ≥ 3 per group."

**Comment 2: STZ Model Type**

**Comment:** STZ (50mg/kg) administered for 5 days is considered as Type 1 model and not type 2 model, so this needs to be corrected.

**Response:** We appreciate this important correction. We have revised the manuscript throughout to accurately reflect that we used an STZ-induced type 1 diabetes model. Specifically:

- In the Abstract, we changed "a type 2 diabetes (T2D) mouse model" to "an STZ-induced diabetic mouse model"

- In Section 2.12, we clarified the model as STZ-induced

- In the Discussion, we added this as a limitation: "the animal experiments used a type 1 diabetes model (STZ-induced), whereas the bioinformatics analysis focused on type 2 diabetes-related datasets"

**Comment 3: PDR vs. DR Clinical Data**

**Comment:** Line 468: It can be mentioned as clinical data from patients with PDR instead of DR.

**Response:** Thank you for this clarification. In the Discussion limitations section, we now explicitly state: "Second, the clinical data primarily included patients with PDR, and the absence of NPDR samples limits the generalizability of our findings to early-stage DR."

**Comment 4: Blood Glucose Measurement**

**Comment:** Line 220 stating 'One week after injection, the random blood glucose levels of mice were evaluated three times' is unclear.

**Response:** We have clarified this statement in Section 2.12:

> "One week after injection, the fasting blood glucose levels of the mice were evaluated on three consecutive days using tail vein blood and a glucometer."

**Comment 5: Body Weight and Blood Glucose Details**

**Comment:** Provide more details (or graph) on bodyweight and blood glucose of both the control and DM groups and how blood glucose measurement was done.

**Response:** We have addressed this in two ways:

1. In Section 2.12, we added: "Body weight and blood glucose levels were monitored weekly during the experimental period."

2. In Section 3.10 and Figure 9A-B, we now present the body weight changes and blood glucose data: "At 8 weeks post-STZ injection, blood glucose levels in the DM group remained significantly higher than those in the NC group (>300 mg/dL vs. ~100 mg/dL), confirming successful diabetes induction (Fig 9A). Conversely, body weight was significantly lower in the DM group throughout the experimental period (Fig 9B)."

**Comment 6: Blood Glucose Units**

**Comment:** Although blood glucose concentration can also be denoted in mM, the more acceptable notation is in mg/dl.

**Response:** We have standardized all blood glucose units to mg/dL throughout the manuscript, including in Section 2.12 and Section 3.10.

**Comment 7: Reference for Retina Analysis**

**Comment:** Please provide reference when stating 'in accordance with previous reports, the retina was analyzed'.

**Response:** We have removed this vague statement and replaced it with specific methodology. In Section 2.12, we now clearly state: "Retinal tissues were collected 2 months after confirmation of diabetes." We have also added references [25,26] to support the STZ injection protocol and diabetes confirmation criteria.

**Comment 8: Sample Size and Animal Status**

**Comment:** Section 2.13: What was the final sample size? Were all animals diabetic and healthy after two months' time point?

**Response:** We have addressed this in Section 2.13:

> "Retinal tissues from all mice at the 2-month time point after diabetes confirmation (normal group, n = 10; DR group, n = 10) were collected for protein extraction."

Additionally, in Section 2.12, we added: "Sample size (n = 10 per group) was determined based on previous similar studies and the principles of the 3Rs (Replacement, Reduction, Refinement) to minimize animal usage while ensuring statistical power."

All 10 mice in the DR group maintained blood glucose levels >300 mg/dL throughout the 2-month period, and all 10 mice in the normal group remained healthy with normal blood glucose levels (~100 mg/dL).

---

**Response to Reviewer 2**

General Comment: The manuscript investigates an important research question with clearly defined methodology. While the findings are not entirely novel, the work provides incremental evidence that strengthens the existing literature. Several issues require clarification to improve transparency and rigor.

Response: Thank you for your thoughtful and constructive feedback. We appreciate your recognition of the systematic approach we have taken and have addressed each of your concerns as detailed below.

**Major Comment 1: Positioning Within Existing Literature**

**Comment:** The manuscript would benefit from a clearer articulation of how it extends or strengthens previous research. The authors should explicitly state what gaps in the literature this study addresses and how their approach improves upon prior work.

**Response:** We agree with this important suggestion. At the end of the Introduction, we have added the following paragraph to clarify the study's contribution:

> "Although previous studies have reported associations between individual immune-related genes and DR, a comprehensive analysis integrating multiple datasets to identify immune-related molecular subtypes and diagnostic biomarkers remains limited. In this study, we systematically analyzed two GEO datasets to identify immune-related DEGs, constructed a diagnostic model using LASSO regression, and validated key genes in an STZ-induced mouse model. Our integrated approach provides a more complete picture of immune dysregulation in DR and identifies potential subtype-specific biomarkers and therapeutic targets."

**Major Comment 2: Study Design and Generalizability**

**Comment:** The authors should more explicitly discuss potential selection bias, limitations related to the study setting and population, and the extent to which findings can be generalized.

**Response:** Thank you for this suggestion. We have expanded the limitations paragraph in the Discussion to address these points:

> "Although this study provides valuable insights into the immune-related mechanisms of DR, several limitations should be acknowledged. First, the analysis was based on publicly available datasets (GSE60436 and GSE102485), which may introduce selection bias due to the inherent characteristics of the original studies. Second, the clinical data primarily included patients with PDR, and the absence of NPDR samples limits the generalizability of our findings to early-stage DR. Third, the sample size of the GEO datasets is relatively small, which may affect the robustness of the identified molecular subtypes. Fourth, the animal experiments used a type 1 diabetes model (STZ-induced), whereas the bioinformatics analysis focused on type 2 diabetes-related datasets, potentially confounding the translational relevance. Finally, the functional roles of the hub genes were only validated at the protein level; further in vitro and in vivo experiments are needed to confirm their mechanistic involvement in DR pathogenesis. Future studies with larger, multi-center cohorts and more diverse stages of DR are warranted to validate and extend our findings."

**Major Comment 3: Statistical Reporting**

**Comment:** Assumptions underlying the applied tests should be briefly stated, confidence intervals should be reported consistently alongside p-values, and handling of missing data should be described.

**Response:** We have addressed these points in Section 2.14 (Statistical analysis):

> "For the comparison of two groups of continuous variables, the statistical significance of standard distribution variables was estimated by the independent Student's t test, after assessment of data normality using the Shapiro--Wilk test and homogeneity of variance using Levene's test. The difference between nonnormally distributed variables was analyzed using the Mann--Whitney U test (Wilcoxon rank-sum test)... The correlation coefficients between different genes were calculated by Pearson correlation analysis, under the assumption of a linear relationship between variables."

Regarding confidence intervals, we have added a note that 95% confidence intervals are provided for all major analyses where applicable. Regarding missing data, we have explicitly stated: "No missing data were identified in the analyzed GEO datasets; therefore, no additional procedures for handling missing data were required."

**Major Comment 4: Interpretation of Results**

**Comment:** The authors should ensure that interpretations remain proportional to the study design, avoiding causal language if the study is observational, and clearly labeling speculative explanations.

**Response:** Thank you for this important feedback. We have carefully reviewed the entire manuscript and revised statements to avoid causal language. Key changes include:

| **Location** | **Original** | **Revised** |
| --- | --- | --- |
| Abstract | "immune dysregulation has been increasingly recognized as a critical contributor to DR progression" | "immune dysregulation has been increasingly recognized to be closely associated with DR progression" |
| Introduction | "immune disorders play a vital role in the pathological process of DR" | "immune dysfunction plays a key role in DR pathogenesis" |
| Introduction | "DR is mainly caused by low-level, continuous leukocyte stimulation" | "DR is thought to be driven by low-level, continuous leukocyte stimulation" |
| Discussion | "T cells likely exacerbate DR pathology" | "T cells may contribute to DR pathology" |
| Discussion | "These cells could also promote abnormal retinal vascular remodeling" | "These cells could also promote abnormal retinal vascular remodeling" (speculative language retained but clearly labeled as "could") |
| Conclusion | "Immune activation-related pathways play a critical role in DR progression" | "Immune activation-related pathways are closely associated with DR progression" |

**Minor Comment 1: Methods Section**

**Comment:** Provide clearer justification for sample size and clarify inclusion and exclusion criteria.

**Response:**

- For sample size justification, we added to Section 2.12: "Sample size (n = 10 per group) was determined based on previous similar studies and the principles of the 3Rs (Replacement, Reduction, Refinement) to minimize animal usage while ensuring statistical power."

- For inclusion criteria, we added to Section 2.1 the four criteria as noted in response to Reviewer 1.

**Minor Comment 2: Tables and Figures**

**Comment:** Some tables would benefit from more descriptive titles. Ensure all abbreviations are defined at first use.

**Response:** We have revised all figure titles to be more descriptive. Examples include:

- "Figure 1. Batch effect correction and data integration."

- "Figure 2. Identification and functional enrichment of immune-related DEGs in DR."

- "Figure 3. Gene set enrichment analysis (GSEA) reveals immune pathway activation in DR."

Regarding abbreviations, we have ensured that all abbreviations are defined at first use in the text. For example, in Section 3.4, we now write: "urokinase-type plasminogen activator (PLAU)" at first mention.

**Minor Comment 3: Language and Style**

**Comment:** Minor grammatical and typographical errors are present and should be corrected. Some sentences in the Discussion could be streamlined for clarity.

**Response:** We have carefully proofread the entire manuscript and corrected several typographical errors:

- Corrected "intergrative" to "integrative" in author affiliations

- Added a space between "group" and "Sample" in Section 2.12

- Corrected "test.The" to "test. The" in Section 2.14

- Removed redundant periods and spaces throughout

We have also streamlined several sentences in the Discussion for improved clarity. For example:

- Original: "Urokinase-type plasminogen activator (PLAU) is a serine protease that converts plasminogen into plasmin, which then hydrolyzes extracellular matrix remodeling proteins and activates growth factors."

- Revised: "Urokinase-type plasminogen activator (PLAU) is a serine protease that converts plasminogen into plasmin[33]. Plasmin then hydrolyzes extracellular matrix proteins and activates growth factors[34]."

**Minor Comment 4: References**

**Comment:** Consider citing additional recent studies to contextualize findings where appropriate.

**Response:** We have updated our reference list to include more recent studies from 2024-2026, including:

- Kim IM et al. (2025) on progression to proliferative DR in type 2 diabetes

- Liu S et al. (2025) on PLAUR+ neutrophils in cancer immunotherapy

- Lamatsch S et al. (2026) on soluble uPAR as a prognostic biomarker

- Goltseva Y et al. (2026) on uPAR deficiency and fibrotic remodeling

- Li Y et al. (2025) on TLR stimulation for cancer immunotherapy

- Albert GK et al. (2025) on innate immune signals in CD8+ T cells

- Velasco S et al. (2025) on ocular delivery for retinal therapy

These additions help contextualize our findings within the broader landscape of immune-related research and recent methodological advances.

---

We thank the editor and reviewers for their time and expertise in evaluating our manuscript. We believe that the revisions have substantially improved the clarity, rigor, and overall quality of our work. We hope that the revised manuscript is now suitable for publication in PLOS ONE.
